# Data Communications Using Guided Elastic Waves by Time Reversal Pulse Position Modulation: Experimental Study

**DOI:** 10.3390/s130708352

**Published:** 2013-07-01

**Authors:** Yuanwei Jin, Yujie Ying, Deshuang Zhao

**Affiliations:** 1 Department of Engineering and Aviation Sciences, University of Maryland Eastern Shore, Princess Anne, MD 21853, USA; 2 Department of Civil and Environmental Engineering, Carnegie Mellon University, Pittsburgh, PA 15213, USA; E-Mail: yyingcmu@gmail.com; 3 School of Physical Electronics, University of Electronic Science and Technology of China, Chengdu 610054, China; E-Mail: deshuangzhao@gmail.com

**Keywords:** piezoelectric sensors, structural health monitoring, communication, telemetry, time reversal, guided elastic waves, timing acquisition, steel pipes

## Abstract

In this paper, we present and demonstrate a low complexity elastic wave signaling and reception method to achieve high data rate communication on dispersive solid elastic media, such as metal pipes, using piezoelectric transducers of PZT (lead zirconate titanate). Data communication is realized using pulse position modulation (PPM) as the signaling method and the elastic medium as the communication channel. The communication system first transmits a small number of training pulses to probe the dispersive medium. The time-reversed probe signals are then utilized as the information carrying waveforms. Rapid timing acquisition of transmitted waveforms for demodulation over elastic medium is made possible by exploring the reciprocity property of guided elastic waves. The experimental tests were conducted using a National Instrument PXI system for waveform excitation and data acquisition. Data telemetry bit rates of 10 kbps, 20 kbps, 50 kbps and 100 kbps with the average bit error rates of 0, 5.75 x 10-^4^, 1.09 x 10-^2^ and 5.01 x 10-^2^, respectively, out of a total of 40, 000 transmitted bits were obtained when transmitting at the center frequency of 250 kHz and a 500 kHz bandwidth on steel pipe specimens. To emphasize the influence of time reversal, no complex processing techniques, such as adaptive channel equalization or error correction coding, were employed.

## 1. Introduction

Piezoelectric sensor networks that are embedded in large civil structures, such as pipes, offshore platforms, bridges and railways, are considered as the future of structural health monitoring (SHM) systems [1]. Active acoustic sensors or actuators in the network can be excited to generate elastic waves that could propagate a long distance and interrogate structures for possible defects, thus providing *in situ* monitoring of the integrity and operating conditions of the structures. One of the important aspects of sensor networks for SHM applications is its ability for data communication between sensors. This is because the operating status and conditions of the structure under monitoring must be sent through the network to a central processing unit, so that human operators can take necessary actions when defects are detected. Data communication within a sensor network could occur between two or multiple sensor nodes or between sensor nodes and a central processing unit. In the classic communication theory, information-bearing signals are modulated and excited from a transmitter; the excitation signals propagate through physical medium as propagation waves, such as electromagnetic waved and acoustical waved, and are received by a receiver. The received signals are demodulated to recover the transmitted information bits. In many existing SHM sensor network applications, conventional schemes, such as wireless radio communication and acoustical communication, are employed for data communication. However, the underlying wave propagation physics reveals that conventional communication schemes are often inapplicable or inadequate in many real-life situations. For example, for pipes that are buried underground or for hollow sub-sea structures in offshore platforms, radio communication is difficult to realize, because electromagnetic wave signals suffer severe decay in soil or water, which results in very short transmission range.

Ultrasonic waves, *i.e.*, waves that travel along a rod, tube, pipe or plate-like structure, form guided waves as a result of the interaction between ultrasonic excitation signals propagating in a medium and the medium's boundaries [2-4]. The excitation of guided ultrasonic waves has become a popular tool for nondestructive inspection of pipes and other physical infrastructures, due to their potential to travel great distances [5-8]. However, there are three fundamental difficulties when guided waves are utilized for pipe inspection and data communication, *i.e.*, signal dispersion, multimode and ambient noise. First, the elastic guided wave signals are highly dispersive. Signal dispersion stems from the underlying elastic wave propagation physics, which signal transmission speed varies with frequencies. Acoustic signal dispersion causes signal time spreading, as well as phase and frequency shift. In communication theory, this phenomenon is called multipath propagation. The delayed arrivals of propagating signals may overlap, destructively causing severe signal attenuation or signal fading, which results in poor system performance in many stages of the communication flow, including synchronization, signal detection and demodulation, *etc.* Second, pipe waves are characterized by an infinite number of dispersive longitudinal and torsional modes and a doubly infinite number of flexural modes [9]. The longitudinal and torsional modes are both axisymmetric, while each flexural mode exhibits an infinite number of non-axisymmetric circumferential mode orders [10,11]. Thus, a proper selection of wave modes for signal transmission would be a critical, but difficult, task. Third, ambient noise exists in the received signals. Ambient noise refers to the addition of a random signal of specific mean value and variance. A high noise level reduces the signal-to-noise power ratio at the receiver, which yields poor performance for signal detection. Adaptive filtering methods should be employed to reduce the level of ambient noise.

Time reversal has emerged as a viable approach for overcoming signal dispersion for digital communication applications, for example, underwater acoustic communication [12] and ultra-wideband radio communication [13–16]. In [17,18], we develop a time reversal-based guided elastic wave communication scheme that utilizes steel pipes as transmission channels. The developed communication scheme was verified by preliminary experimental data collected from a steel pipe in a laboratory environment using acoustic wave excitation. The focus of this paper is to provide a comprehensive theoretical development of the proposed time reversal-based pulse position modulation (TR-PPM) method and conduct extensive experimental tests to examine the achievable bit error rate (BER) on steel pipe specimens with various configurations using lead zirconate titanate (PZT) wafer transducers. The contribution of the work described in this paper and our earlier papers [17,18] focuses on the following two perspectives.

First, we utilize the time reversal-based pulse position modulation (TR-PPM) method to achieve signal synchronization and symbol demodulation in order to compensate for severe channel dispersion and multi-modality in the elastic wave communication system design. In most communication systems, the receiver must know the exact starting time in order to demodulate the transmitted symbols correctly. The task for determining the starting time of symbols at the receiver from the distorted transmission signals is commonly called timing acquisition or synchronization. For high data rate communication using elastic waves, rapid and precise timing acquisition becomes very difficult for the PPM scheme, due to severe dispersion and the multi-modality of the underlying characteristics of elastic waves. This problem is somewhat similar to the ultra-wideband (UWB) communication systems, where a large number of resolvable paths in UWB channels may cause many possible starting times [19–22]. In such cases, timing acquisition often requires complicated searching algorithms to estimate the start time for symbol demodulation. Hence, the hardware implementation is costly. The TR-PPM scheme we proposed in our earlier paper [17,18] and this paper can be regarded as a channel-assisted timing algorithm, which does not require complicated channel estimation algorithms or circuits at the transmitter and the receiver. In our system, the physical channel is implicitly pre-equalized by the time reversed waveforms at the transmitter. By utilizing the intrinsic adaptive space-time focusing of time reversal, the proposed method needs only a small number of training preamble symbols performing in a pitch-catch mode to estimate the starting time of the symbols quickly, thus enabling subsequent symbol demodulation, despite the above-mentioned channel dispersion and multi-modality in elastic wave channels.

Second, we conduct extensive test-bed studies in a laboratory environment to validate the feasibility of elastic wave data communication. The test bed includes PZT wafers, two different steel pipe specimens (*i.e.*, a carbon steel pipe and a stainless steel pipe) and a National Instrument PXI acoustical measurement system that includes an arbitrary waveform generator and a digitizer. We note that data communication schemes using elastic waves have been reported for, for example, flood detection in oil rigs (see, e.g., [23,24]), drill string telemetry [25] in the measurement while drilling (MWD) and logging while drilling (LWD) services in the oil and gas industry [26,27] or Lamb wave communication (see e.g., [28]); systematic studies of high data rate communication schemes using elastic waves are lacking. We note that a pulse position modulation scheme using guided acoustic waves for flood detection was proposed in [23,24]. However, the realized data rate was relatively low, because only a 2 kHz narrow frequency band (39 kHz-41 kHz) was used in order to overcome the problem of channel dispersion. To achieve high data rate communication using guided elastic waves, a much wider frequency band must be used. We test the proposed TR-PPM modulation scheme under the data rates of 10 kbps, 20 kbps, 50 kbps and up to 100 kbps. The obtained average bit error rates are 0, 5.75 x 10-^4^, 1.09 x 10-^2^ and 5.01 x 10-^2^, respectively, out of a total of 40, 000 transmitted binary bits (*i.e.*, ′0′s and ′1′s) when transmitting at the center frequency of 250 kHz and a 500 kHz bandwidth on steel pipe specimens. We note that no complex processing schemes, such as adaptive channel equalization and error correction coding, are employed at the receiver for the purpose of emphasizing the advantage of time reversal.

The remainder of this paper is organized as follows. Section 2 describes the background theory. Section 3 presents the communication results by experiments. The conclusion is presented in Section 4.

## 2. Background Theory

### 2.1. General Signal Models for Pulse Position Modulation

For wideband guided elastic signals, we adopt a signaling method that is similar to the ultra-wide-band (UWB) signaling in radio communications [19,22,29]. A general signaling model can be described as follows. Every transmitted symbol is composed of *N_f_* repeated pulses, called frame. Let *T_s_* and *T_f_* denote the symbol duration and frame duration, respectively. Then, *T_s_= N_f_T_f_*, which means there is one pulse per frame. Each guided elastic pulse, *g(t)*, is of a short duration, T_0_ < *T_f_*, at the microsecond scale, occupying a wide bandwidth of a few hundred kilo-hertz. Each frame contains *N_c_* chips, each of duration, *T_c_*. A general wideband symbol signature waveform transmitted during the acquisition process for a single user can be expressed as a series of pulses:

(1) $$g_{s}(t)=\sum_{j=0}^{N_{f}-1}g(t-{jT}_{f}-c_{j}T_{c})$$

where *c_j_* ∈ {0,1, 2, …, *N_c_* − 1}, ∀j ∈ [0, *N_f_*− 1] represents user-specific pseudorandom time hopping (TH) code for user separation [29], which may take on different patterns, such as fast hopping or slow hopping. The pulse amplitude is scaled to satisfy ∫ *g^2^*(*t*)*dt = 1/N_f_*, such that the symbol waveform has unit energy, $\int g_{s}^{2}(t)dt=1$. Furthermore, the data symbols are modulated during transmission. In pulse-position modulation (PPM), different symbols are realized by shifting a pulse to distinct positions in time within the specified symbol duration. For a binary PPM, the modulated symbols transmitted by the desired user can be described as:

(2) $$s(t)=\sum_{i=-\infty }^{\infty }g_{s}(t-{iT}_{s}-a_{i}\Delta )$$

where a_i_ ∈ {0,1} represent the data symbols that are modeled as binary-independent and identically distributed (i.i.d.; random variables and Δ represents the time shift imposed on all the elastic pulses of a given transmission stream by a unit data symbol.

Next, we discuss the elastic wave propagation channel for wideband signaling. Under the linear elastic wave theory, the overall channel response can be modeled by a stochastic tapped delay line:

(3) $$h(t)=\sum_{l=0}^{L-1}\alpha_{l}f_{l}(t-\tau_{l})$$

where *L* is the number of taps in the channel response, α_l_ is the path gain at excess delay, τ_l_, corresponding to the l-th propagation path. We assume that the delay is *τ*_0_*<* τ_1_*<* … *<* τ_L___1_. The first arrival, *τ*_0_, is normally referred to as the timing information and is measured with reference to the receiver's local clock for demodulation purposes. The function, *f_l_*(*t*), represents the combined responses of the transmit PZT sensor, the receive PZT sensor, as well as the propagation channel corresponding to the *l*-th path of a transmitted pulse. We note that in this paper, the underlying assumption regarding a surface-mounted PZT wafer active sensor is that its response is characterized as a linear time invariant system and, thus, is absorbed in *f_l_*(*t*). More detailed modeling of PZT wafer active sensors can be found in various literature (see, e.g., [30,31]). For simplicity, we let *f_l_*(*t*) *= δ*(*t*), an ideal channel impulse response. Hence, we can re-write Equation (3) as:

(4) $$h(t)=\sum_{l=0}^{L-1}\alpha_{l}\delta (t-\tau_{l,0}-\tau_{0})$$

where τ*_l_*,_0_ ≜ τ_l_ − τ_0_ is the relative time delay of the *l*-th path. The received signal from a single user can then be written as:

(5) r(t)=s(t)*h(t)+w(t)

(6) $$=\sum_{i=-\infty }^{\infty }\sum_{l=0}^{L-1}\alpha_{l}g_{s}(t-iT_{s}-a_{i}\Delta -\tau_{l})+w(t)$$

(7) $$=\sum_{i=-\infty }^{\infty }\hat{s}(t-iT_{s}-a_{i}\Delta -\tau_{0})+w(t)$$

where * denotes linear convolution and ŝ(t) = *g_s_*(*t*) * *ĥ*(*t*) is the received waveform corresponding to a single symbol waveform, where:

(8) $$\hat{h}(t)=\sum_{l=0}^{L-1}\alpha_{l}\delta (t-\tau_{l,0})$$

is a pseudo-channel response by eliminating the first arrival of *h(t)*. The disturbance term, *w(t)*, is assumed as a wide sense stationary process characterized as the additive noise with zero-mean and complex Gaussian distribution.

The symbol, τ_0_, is the direct propagation delay between the transmitter and the receiver and can be expressed as τ_0_ = *n_s_T_s_* + *n_f_T_f_* + ε, based on different time scales at the symbol-level, frame-level and pulse-level [19]. Here, *n_s_=* ⌊τ_0_*/T_s_*⌋ stands for the symbol level offset, *n_f_=* ⌊ (τ_0_− *n_s_T_s_*)/ *T_f_*⌋ is the frame-level offset, where *n_f_* ∈ [0, *N_f_−* 1], and the pulse level offset is ε ∈ [0, *T_f_)*. ⌊*x*⌋ represents the largest integer not greater than *x*. Typically, a symbol-by-symbol sliding correlation filer is applied to generate samples, x[*i* x], at the symbol rate by [19,22,32]:

(9) $$x[i]=\int_{0}^{T_{s}}r(t+iT_{s})pr(t-\tau_{0})dt$$

where *p_r_* (*t*) is the correlation template. Under the ideal case, where the channel knowledge is known and the timing, τ_0_, is accurate, we choose *p_r_(t) = g_s_(t)*, which is equivalent to maximum ratio combining, and Equation (9) is the optimal detector for demodulation. Typically, τ_o_ is unknown and needs to be estimated. The accuracy of the timing estimate and the choice of the template, *p_r_*(*t*), would significantly affect the received energy of the sample, *x* x[*i* x], which, in turn, impacts directly the bit error rate. The timing acquisition problem for guided elastic wave communication is challenging in particular, due to the inherent propagation characteristics of the elastic channel. Elastic waves are dispersive, which implies that there exists severe multipath energy spreading; Elastic waves are also multi-modal, which means strong peaks of the received signals may appear in many locations of the time domain waveform trace. To overcome these problems in timing acquisition and symbol demodulation, we propose the time reversal PPM method in the next sub-section. For simplicity, we choose *N_c_=* 1 and *N_f_* = 1. Hence, *T_s_= T_f_* and the general model of PPM in Equations (1) and (2) is therefore simplified.

### 2.2. Description of Time Reversal PPM

The principle of time reversal for acoustic applications originates from the invariance of the governing wave equations in underwater acoustics [33] and in guided acoustic waves [34-37]. Under the invariance principle, if *u*(*r,t*) is the solution to the wave equation, the time reversed version of the original solution, *i.e., u*(*r, −t*), also satisfies the wave equations. This observation implies that if we record the waves radiated from a certain source and retransmit their reversed waves back to the same medium, the retransmitted waves will retrace their incoming paths back towards the original source. Time reversal has been successfully applied to ultra-wideband electromagnetic wireless communication systems of various configurations [13,15,38,39]. In this paper, we consider a single-transmitter and single-receiver configuration in a pitch-catch mode for elastic wave communication. The time reversal method for timing acquisition can be summarized in the following four steps:

Step (1) **Channel probing (B***→***A).** In this step, a pilot elastic wave pulse, *p*(*t*), is initiated from the intended receiver, B, for probing the multipath propagation channel between B and the intended transmitter, A. The received signals at A can be expressed by:
  (10) y(t)=p(t)*h(t)
  where *h*(*t*) is the impulse response of the elastic wave channel coupled with the transmit and receiver piezoelectric transducers, which is typically very dispersive, due to the intrinsic properties of elastic waves. Note that the additive noise is omitted for simplicity purposes.
Step (2) **Time reversal.** The received signal is time reversed in the time domain. The time reversing operation is equivalent to reading the data by first-in-last-out, which yields:
  (11) y(−t)=p(−t)*h(−t)
  Here, *y*(−*t*) is called the time reversed waveform. Note that by the temporal and spatial focusing characteristics of time reversal, if we retransmit *y*(−*t*) back to the channel and assume that the channel reciprocity holds, the retransmitted waveform will focus the scattered multipath energies on the initial source receiver temporally and spatially. Thus, the intensity of the signal received by the receiver is not only enhanced greatly, but the time spread is also compressed effectively. The validity of the focusing effects have been studied in elastic wave theory in [34-37].
Step (3) **Preambles transmission for timing acquisition (A***→***B)** In time-reversal-based timing acquisition, we use the time reversed waveform, *y*(−*t*), in Equation (11) as the basic signal waveform for carrying the timing preambles. The main advantage of using the time reversed waveform as the information carrier is that complicated channel estimation algorithms are not necessary at the transmitter, because the channel information is inherently included in the time reversed waveform. Thus, the transmitting waveforms take the form of:
  (12) $$s_{p}(t)=\sum_{n=0}^{N-1}k\cdot y(-t+nT_{f}),0\leq NT_{f}$$
  where *T_f_* is the frame duration for one bit transmission, *N* is the number of preamble training bits and *k* is the energy normalization factor to ensure the transmission energy remains unchanged for each symbol.
Step (4) **Data symbol transmission (A***→***B)** Following the preamble sequences, the data symbols are transmitted. Here, we use a pulse position modulation scheme, which takes the form of:
  (13) $$s_{d}(t)=\sum_{j=0}^{M-1}k\cdot y(-t+jT_{f}+a_{j}\Delta ),t>NT_{f}+T_{g}$$
  where Δ is the shifting time for distinguishing the pulses carrying bit ‘0′ or bit ‘1′. The guard time, *T_g_*, is pre-determined and is inserted between the training preamble symbols and the data symbols for the purpose of preventing the data waveforms from overlapping the timing preambles. All binary data, *a_j_* ∈ {0,1}, are modulated by the pulse position modulation (PPM; scheme, where *M* is the total number of binary bits for each data package to be transmitted.

### 2.3. Timing Acquisition by Time Reversal

Next, we estimate the starting time of the preamble training sequences at the receiver. After the transmitted signals, *s_p_(t)*, propagate through the channel, the preambles arriving at the receiver become:

(14) $$r_{p}(t)=\sum_{n=0}^{N-1}y(-t-nT_{f})*h(t)=\sum_{n=0}^{N-1}p(-t+nT_{f})*h(-t)*h(t)$$

Note that we omit the scaling factor, *k*, given in Equation (12) in the derivation, because it approximates a constant for static channels. It is well known that by time reversal, *h*(−*t*) * *h*(*t*) is the auto-correlation function of *h*(*t*), which appears to be a highly focused waveform when *h*(*t*) is dispersive. Here, we employ only one sliding correlation detector combined with a single tap finger to capture and detect the received signals. To recover the starting time from the received preambles, a locally generated template, *u*(*t*), is applied to matching them in a sliding mode as follows:

(15) $$R_{p}(\tau )=\int_{0}^{NT_{f}}r_{p}(t)\cdot u(t-\tau )dt$$

(16) $$=\int_{0}^{NT_{f}}\left(\sum_{n=0}^{N-1}p(-t+nT_{f})*h(-t)*h(t)\right)\cdot u(t-\tau )dt$$

where $u(t)=\sum_{n=0}^{N-1}p(t-nT_{f})$. Inserting the elastic wave propagation channel model Equation (4) into Equation (16) yields:

$$R_{p}(\tau )=\int_{0}^{NT_{f}}\left(\sum_{n=0}^{N-1}p(-t+nT_{f})*\sum_{i=0}^{L-1}\alpha_{i}\delta (-t-\tau_{i})*\sum_{j=0}^{L-1}\alpha_{j}\delta (-t-\tau_{j})\cdot \sum_{m=0}^{N-1}p(-t-mT_{f}-\tau )\right)dt$$

Next, we let the autocorrelation function be $R_{pp}(\tau )=\int_{-\infty }^{+\infty }p(-t)p(t-\tau )dt$ and Δτ_ij_ = *τ_j_− τ_i_*, Δ*T*_mn_ = (*n − m*)*T_f_*; we can re-write *R_p_*(*τ*) as:

(17) $$\begin{array}{cc} R_{p}(\tau ) & =\sum_{i=0}^{L-1}\sum_{j=0}^{L-1}\sum_{n=0}^{N-1}\sum_{m=0}^{N-1}\alpha_{i}\alpha_{j}R_{pp}(\tau -\Delta \tau_{ij}-\Delta T_{mn})\\ & =Q_{0}(\tau )+Q_{1}(\tau )+Q_{2}(\tau )+Q_{3}(\tau ) \end{array}$$

where:

(18) $$Q_{0}(\tau )=\sum_{i=j=0}^{L-1}\sum_{n=m=0}^{N-1}\alpha_{i}^{2}R_{pp}(\tau )=NR_{pp}(\tau )\sum_{i=0}^{L-1}\alpha_{i}^{2}$$

is the term that represents the autocorrelation between the multipath components. The rest of the three terms in Equation (17) are the cross-correlation terms:

(19) $$Q_{1}(\tau )=\sum_{i=j=0}^{L-1}\sum_{n\neq m}^{N-1}\sum_{m\neq n}^{N-1}\alpha_{i}^{2}R_{pp}(\tau -\Delta T_{mn})$$

(20) $$=\sum_{i=0}^{L-1}\alpha_{i}^{2}\sum_{n\neq m}^{N-1}\sum_{m\neq n}^{N-1}R_{pp}(\tau -\Delta T_{mn})$$

(21) $$Q_{2}(\tau )=\sum_{i\neq j}^{L-1}\sum_{j\neq i}^{L-1}\sum_{n=m=0}^{L-1}\alpha_{i}\alpha_{j}R_{pp}(\tau -\Delta T_{ij})$$

(22) $$Q_{3}(\tau )=\sum_{i\neq j}^{L-1}\sum_{j\neq i}^{L-1}\sum_{n\neq m}^{N-1}\sum_{m\neq n}^{N-1}\alpha_{i}\alpha_{j}R_{pp}(\tau -\Delta \tau_{ij}-\Delta T_{mn})$$

Since the autocorrelation collects all the signal energies of the multipath components of the received preambles, *Q*_0_(*τ*) has the strongest intensity and possesses the most energy of the detector output. As a result, the last three terms can be neglected, because the cross-correlation results in destructive addition between the template and the received preambles. Thus, we have:

(23) $$Q_{0}(\tau )\gg Q_{1}(\tau ),Q_{0}(\tau )\gg Q_{2}(\tau ),Q_{0}(\tau )\gg Q_{3}(\tau )$$

We emphasize that the approximation given in Equation (23) has been verified using experimentally measured elastic signals. A detailed theoretical performance analysis regarding the impact of the approximation is out of the scope of this paper and will be discussed elsewhere. As a result of the approximation, Equation (17) can be simplified as:

(24) $$R_{p}(\tau )=\sum_{i=0}^{L-1}\sum_{n=m=0}^{N-1}\alpha_{i}^{2}R_{pp}(\tau )=N\sum_{i=0}^{L-1}\alpha_{i}^{2}R_{pp}(\tau )$$

Finally, the start time of the preamble symbols is determined by searching the peak of the output of the sliding correlation detector in Equation (24) as follows:

(25) $${\hat{\tau }}_{0}=\underset{0\leq \tau \leq NT_{f}}{\arg\max}\{R_{p}(\tau )\}=\underset{0\leq \tau \leq NT_{f}}{\arg\max}\left\{N\sum_{i=0}^{L-1}\alpha_{i}^{2}R_{pp}(\tau )\right\}$$

Once the starting time of the preamble symbols, *τ̂*_0_, is determined, the receiver performs the demodulation by choosing:

(26) $$t_{0}≜T_{g}+{\hat{\tau }}_{0}$$

as the start of the data symbols.

### 2.4. Signal Detection and Demodulation

The estimate of the timing, *t*_0_, is then utilized for signal demodulation using either non-coherent detectors, such as energy detector (ED) and peak detector (PD) or a coherent detector (CD). For low system complexity and power consumption, we focus on the noncoherent reception of PPM signals [32], which is a generalized likelihood detection. The symbol decision is based on finding the pulse position that contains the maximum energy. The symbol-by-symbol detection process does not require the estimation of the channel parameters. The energy of the multipath components is collected to increase the detection probability of the actual transmitted pulse. For binary PPM, we first calculate two energy quantities:

(27) $$E_{0}≜\int_{-\frac{T}{2}}^{\frac{T}{2}}r^{2}(t-t_{0})dt,E_{1}≜\int_{-\frac{T}{2}}^{\frac{T}{2}}r^{2}(t-t_{0}-\Delta )dt$$

where *r*(*t*) is the the received focused pulse for demodulation. *T* is the time window for energy integration. The choice of *T* depends on the time support, *T_s_*, of the initial probe pulse, *p*(*t*). In practice, one can choose *T = T_s_* or a faction of *T_s_*, such that the majority (e.g., 99%) of the signal energy is retained, (*i.e.*, $\int_{-T/2}^{T/2}p^{2}(t)dt/\int_{-T_{s}/2}^{T_{s}/2}p^{2}(t)dt=0.99$). Thus, the data symbols can be detected using the energy detector by:

(28) $$\{\begin{array}{cc} \text{Symbol}\prime 0\prime \text{detected} & \text{if}\;E_{0}>E_{1}\\ \text{Symbol}\prime 1\prime \text{detected} & \text{if}\;E_{0}<E_{1} \end{array}$$

Assuming additive white Gaussian noise with power spectrum density, *N*_0_, the bit error rate (BER) can be calculated by [32]:

(29) $${\text{BER}}_{\text{ED}}=\frac{1}{2}\text{erfc}\;\left(\frac{E_{p}}{2(N_{0}+N_{1})}\right)$$

where the complementary error function, $\text{erfc}(x)=\frac{2}{\sqrt{\pi }}\int_{x}^{\infty }e^{-t^{2}}dt$. The signal energy and the inter-symbol interference (ISI) are given by:

(30) $$E_{p}=\int_{-\frac{T}{2}}^{\frac{T}{2}}r^{2}(t-t_{0})dt$$

(31) $$N_{1}=\int_{-\frac{T}{2}}^{\frac{T}{2}}{\left(\sum_{k=-K,k\neq 0}^{k=K}r(t-kT-t_{0})\right)}^{2}dt$$

respectively, where ***K*** is the maximum number of the modulated pulses whose tails overlap the focusing pulse. Note that if we choose *T* = ε, where ε stands for a very small time interval, the energy detector approximates a peak detector (PD). The peak detector searches for the focusing peak of the received signals in a time window of duration, T, once the starting time, t_0_, is obtained. Next, the corresponding time, *t*_PD_, when the focusing peak occurs is then identified and is used to demodulate the PPM signals.

## 3. Communication Results by Experiments

In this section, we will discuss the experimental configuration and the obtained results conducted on steel pipes in a laboratory using the time reversal pulse position modulation communication scheme.

### 3.1. Experimental Setup

**Figure 1.**
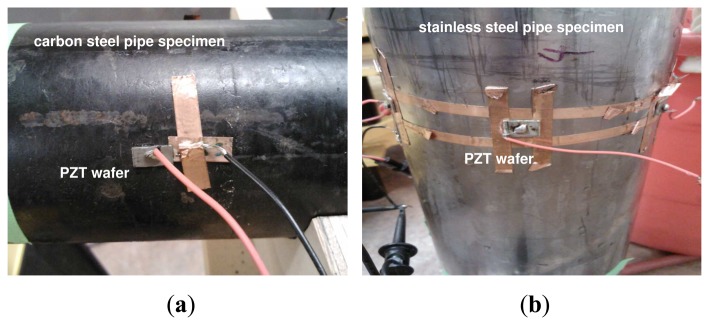
Lead zirconate titanate (PZT) wafers mounted on the surface of two pipe specimens. (**a**) A carbon steel pipe; (**b**) a stainless steel pipe.

Figure 1 shows the steel pipe specimens and the acoustic sensors that were used in experiments. Two sets of experimental configurations were employed. The first set of experiments included a carbon steel pipe specimen of 1.833 meters in length, 70 mm in outside diameter and 4 mm in wall thickness (see Figure 1(a)) and two PZT wafers (PSI-5A4E, Piezo Systems, Inc., Cambridge, MA) that were mounted on the surface of the carbon pipe using cyanoacrylate adhesive, located 1.5 meters apart. Each wafer was 12 mm long and 6 mm wide. The second set of experiments included a stainless steel pipe of 1.5 meters in length, 115 mm in outside diameter and 6 mm in wall thickness (see Figure 1(b)). On the stainless steel pipe, two PZT wafers (PSI-5A4E) were mounted on the surface of the pipe, also using cyanoacrylate adhesive, located 0.69 meters apart. Each wafer was 10 mm long and 10 mm wide.

**Figure 2.**
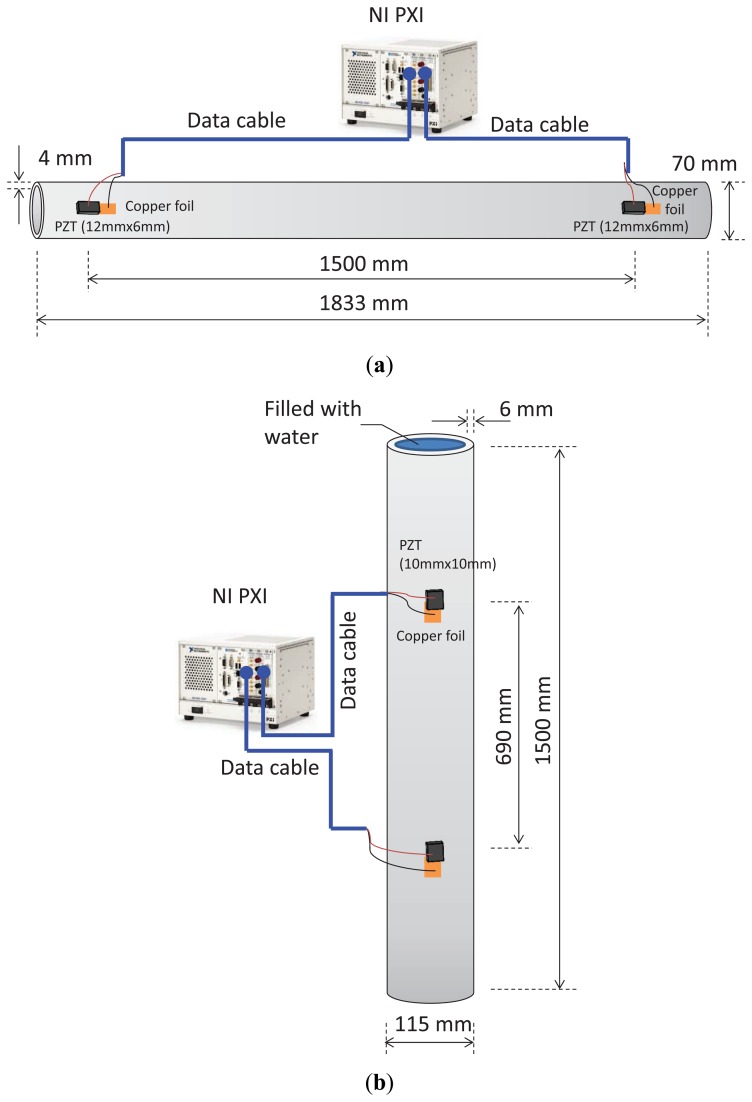
Illustration of experimental setup with cable connections and circuit wiring. (**a**) A carbon steel pipe specimen; (**b**) a stainless steel pipe specimen. Water can be filled into the pipe.

The signal transmission and data acquisition unit was a National Instruments (NI) PXI system, which consists of an arbitrary waveform generator (AWG), a digitizer/oscilloscope and a controller. The waveform generator had a sampling rate 100 MS/s and a 16-bit resolution. The AWG can generate arbitrary waveforms in response to the dispersive medium, thus implementing time reversal. The digitizer had a sampling rate of 100 MS/s and a 14-bit resolution. The waveform generator drives the transmission of the PZT sensor. The digitizer was connected to the receiving PZT sensor and records waveforms. The recorded waveforms were processed online using LabView programming for symbol demodulation and detection. For simplicity, a peak detector was used. The controller synchronizes the operations of the waveform generator and the digitizer. The experimental setup is illustrated in Figure 2. Figure 2(a) depicts the cable connection between the PXI system with a carbon steel pipe specimen. Figure 2(b) depicts the connection for a stainless steel pipe specimen. Water can be filled into the pipe for testing.

### 3.2. Description of Experiments

We follow the four steps given in sub-section 2.2 to conduct experiments.

**Figure 3.**
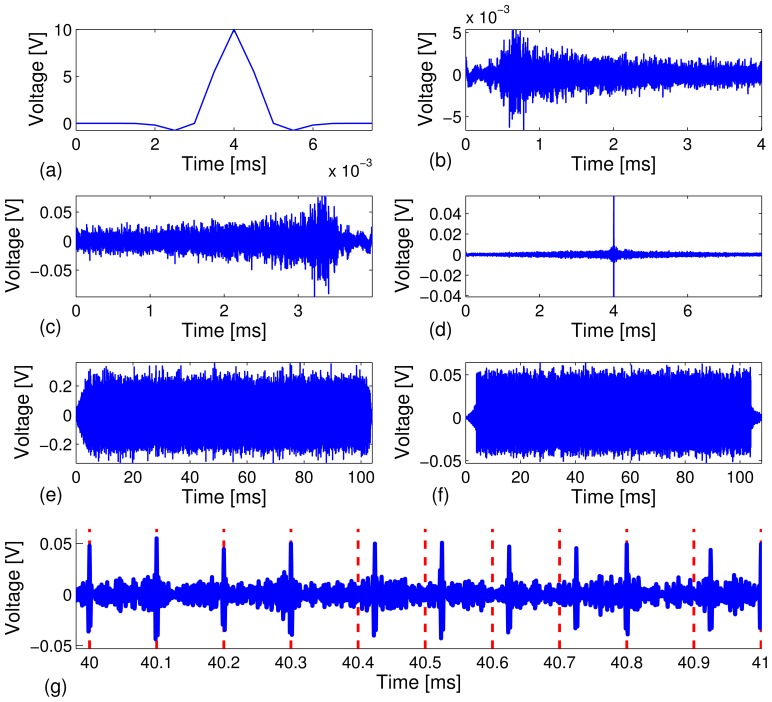
Snapshots of waveforms obtained experimentally from time reversal-based pulse position modulation (TR-PPM) data communications at a data rate of 10 kbps. (**a**) Gaussian pilot signal of 8 *μs*; (**b**) received response after channel sounding; (**c**) time reversal transmission; (**d**) received time reversal focused waveform; (**e**) modulated time reversal PPM waveform; (**f**) received information carrying waveforms; and (**g**) zoomed-in plot of (**f**). The red dashed lines indicate the starting positions of symbol frames. The recovered bit stream in the time window of 40 ms - 41 ms is x[0 0 0 0 1 1 1 1 0 1 x].

**Figure 4.**
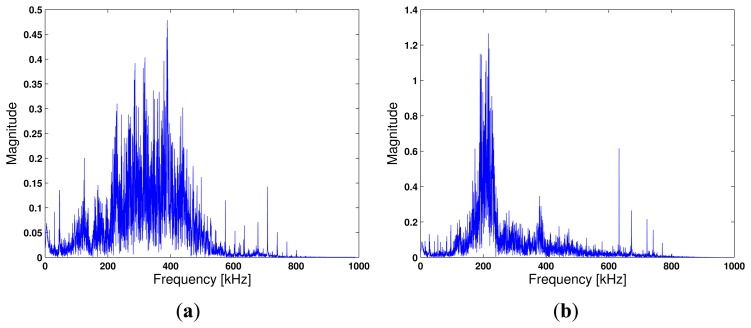
Frequency spectrum of channel responses that include PZT wafers. (**a**) Carbon steel pipe specimen; (**b**) stainless steel pipe specimen.

Step 1: *Channel sounding*. In this step, we choose a Gaussian modulated pulse:
  (32) $$p(t)=\text{Aexp}\left\{-{\left(\frac{t-t_{s}}{\tau_{p}}\right)}^{2}\right\}\cos(2\pi f_{c}t)$$
  as the probe signal, where *f_c_* is the center frequency, τ_p_ = 2 *μs*, which translates to the nominal pulse width of $T_{s}=\sqrt{2}\pi \tau_{p}=8\mu s$ [40], and *t_s_* is the time shift from *t* = 0 when the pulse is generated. *A* is the amplitude (see the example in Figure 3(a)). The total transmission energy is defined as $E_{p}=\int_{-\infty }^{\infty }{\left|p(t)\right|}^{2}dt$. We choose this modulated Gaussian signal as the probe signal for spectral control. The excitation signal has a center frequency, *f_c_* = 250 kHz, with the signal bandwidth of 500 kHz. The signal is amplified by a factor of 10 using a built-in amplifier in the NI's data acquisition system. The maximum voltage of the Gaussian pulse is *A* = 10 volts. The sampling frequency is chosen to be at 2 MHz, four times as much as the maximum frequency of the modulated Gaussian pulse, in order to reconstruct the sampled digital signal into the analog waveform in high fidelity.
  We note that PZT wafers have been explored with continuous sinusoidal or pulsed excitation for defect detection in various applications. Optimal operations of PZT wafers have been studied by many researchers (e.g., [41]), including the procedure to determine the optimal frequency range [42] and analysis of its sensitivity to damage detection [43]. Our data communication experiment was performed by using a nominal frequency bandwidth of 500 kHz. Compared to narrowband excitation, wideband pulse excitation results in more modes and more dispersion [44]. For PZTs with different sizes and coupling conditions, the optimal excitation frequency that results in the highest intensity of the reflected elastic waves may vary. The excitation frequency range (*i.e.*, 0-500 kHz) we chose would cover the optimal frequency for the PZT sensors observed in the experiment. Figure 4(a,b) depict the channel frequency response of the carbon steel pipe specimen and the stainless steel pipe, respectively. The channel frequency response includes the frequency response of the PZT wafers and the propagation channel. Figure 4(a) shows that the peak response of the spectrum falls in the 500 kHz range, while Figure 4(b) shows that the peak response is located at about 200 kHz, which could be caused by the resonance frequency of the PZT wafer.
Step 2: *Time reversal*. As expected, the channel response, *h*(*t*), that characterizes the elastic wave propagation between two positions on a steel pipe is highly dispersive. To realize time reversal, the received signal is time-reversed, energy-normalized and then fed back to the wave generator. We let *y*(*t*) denote the received signal; then, the time reversed signal is *y*(*-t*), as in Equation (11). Note that energy normalization *ofy*(*-t*) is necessary to ensure that the newly constructed transmission waveform has the same energy as the Gaussian pulse transmission used in the channel sounding experiment. The length of the received signal after time-reversal transmission at the digitizer is twice as much as the length of the received signal attained from *Step 1*.
Step 3 & 4: *Data transmission via TR-PPM*. Timing acquisition is not conducted in our experiment, because the PXI system synchronizes the transmission and reception process between the arbitrary waveform generator and the digitizer. A fixed time offset can be determined. Here, we focus on data transmission. The time reversed signal, *y*(*-t*), obtained in Step 2 is employed as a basic waveform for information data transmission. The information data are a stream of binary digits (*i.e.*, zero's and one's) and are coded by the pulse position modulation scheme, which takes the form of:
  (33) $$s(t)=\sum_{j=0}^{M-1}ky(-t)\delta (t-{jT}_{f}-c_{j}\Delta )$$
  where *c_j_* ∈ {0,1} is the binary data to be transmitted, *T_f_* is the frame time to send one bit data, Δ is the additional time shift to distinguish between the pulses carrying the bit, zero or one. *δ*(*·*) is the Dirac delta function. The energy-normalization coefficient is given by $k=\sqrt{E_{p}/E_{y}}$, where the total energy for signal *y*(*-t*) is given by $E_{y}=\int_{-\infty }^{\infty }{\left|y(-t)\right|}^{2}dt$. In our experiments, the information data was a series of *M* = 1, 000 binary numbers, zero's or one's, randomly generated by Lab View programming. The frame time is determined by the prescribed transmitting data rate, *R*, as follows:
  (34) $$T_{f}=\frac{1}{R}$$
  The tests were carried out at the data rates of 10 kbps, 20 kbps, 50 kbps and 100 kbps, which correspond to the frame time of 100 *μs*, 50 *μs*, 20 *μs* and 10 *μs*, respectively. In order to restore the transmitted bit data, the time shift should be larger than the full width of the single Gaussian pulse. Here, we use one fourth of the frame time as the time shift, *i.e.*, $\Delta =\frac{1}{4}T_{f}$. The parameters for the TR-PPM test are summarized in Table 1. The recorded length is mainly determined by the length of the modulated time reversal signal. Here, we use twice the signal length for signal recording at the digitizer.

### 3.3. Results from Experiment I

We conducted the test on a carbon steel pipe to investigate the reliability of our technique by sending a relatively long stream of bits at different data rates, *R* = 10, 20, 50, 100 kbps, and the feasibility of automatic decoding of the bit streams. A total of 40 independent test runs were performed. Under each test run, *M* = 1,000 binary bits were modulated, transmitted and decoded. Out of the 40,000 recovered information bits, we counted the corrected decoded bits and calculated the bit error rate. Figure 3 depicts snapshots of various signals collected in the experiment.

Figure 3(a) depicts a short 8*μs* Gaussian modulated pulse, *p*(*t*), with amplitude, *A* = 10 volts. The signal, *p*(*t*), is excited through the PZT sensor to generate guided acoustic waves that propagate along the carbon steel pipe. The received signal, *y*(*t*), is recorded by the receive PZT transducer at the other end of the pipe specimen (see Figure 3(b)). We can see from Figure 3(b) that the received signal has a very long series of arrivals as a result of multi-modal and elastic wave dispersion, which typically occurs in a cylindrical shell. Moreover, the attenuation level of the dynamic range of the received signal is on the order of 10-^2^, which translates to about 26 dB/m peak power attenuation from the initial excitation signal to the received signal over a 1.5-meter propagation distance. The severe dispersive channel makes it very difficult to achieve data communications using traditional techniques. By the time reversal principle, a time-reversed response waveform, *y*(−*t*) (see Figure 3(c)), is transmitted through the same dispersive channel and results in a much simpler waveform with a strong focused peak at the center (see Figure 3(d)). Time reversal signal processing effectively focuses the multiple dispersive wave modes and enhances the signal-to-noise ratio. Figure 3(e) depicts a snapshot of TR-PPM modulated information carrying waveforms, which is the superposition of 1, 000 time reversed waveforms (Figure 3(c)) under the data rate of 10 kbps with the total time span of 100 ms. The TR-PPM modulated waveforms were excited through the PZT sensor, transmitted and recorded. We note that the TR-PPM transmission waveform was scaled to avoid saturation of the waveform generator of the PXI system. The received information carrying waveform consists of a series of focused peaks, whose positions within a frame indicate bit stream (see Figure 3(f)). Note that a 5 kHz - 600 kHz bandpass filter was applied during the data acquisition process at the receiving PZT sensor to remove low frequency vibrations and high frequency electrical noise. The filtering also prevented saturation of the PPM waveform, due to DC drift. Figure 3(g) is a zoomed-in plot of Figure 3(f) in the 40 ms - 42 ms time window, which includes the decoded 10-bit stream [0 00011110 1].

**Figure 5.**
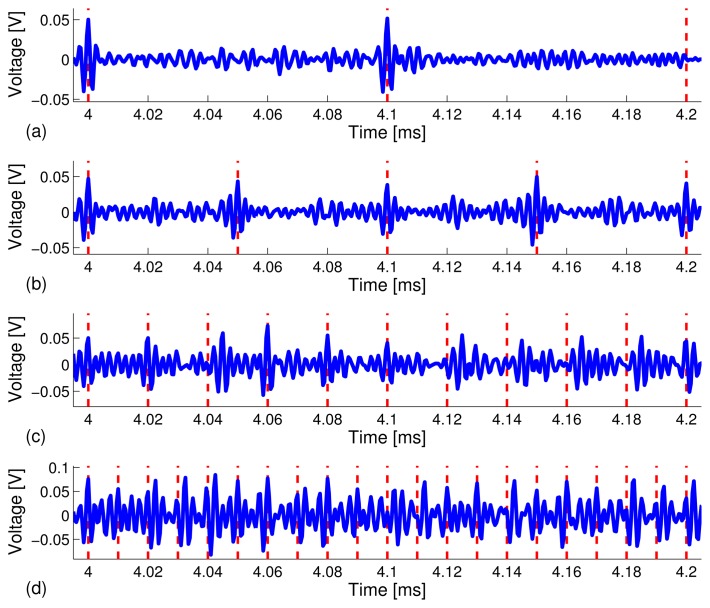
Snapshots of the received information carrying waveforms in the time window of 4.0 ms–4.2 ms. The data rates are: (**a**) 10 kbps; (**b**) 20 kbps; (**c**) 50 kbps; and (**d**) 100 kbps.

Figure 5 depicts snapshots of the zoomed-in view of the waveforms in the 4.0 ms - 4.2 ms time window under different data rates. The peaks are more clearly distinguishable at the lower data rates. The results are what we expected in that a higher data rate, *R*, means a shorter frame time, *T_f_*, which gives rise to higher sideband levels (*i.e.*, interference), due to leakage from adjacent coded waveforms. Thus, the focused peaks become less distinguishable, which results in a higher bit error rate (BER).

**Figure 6.**
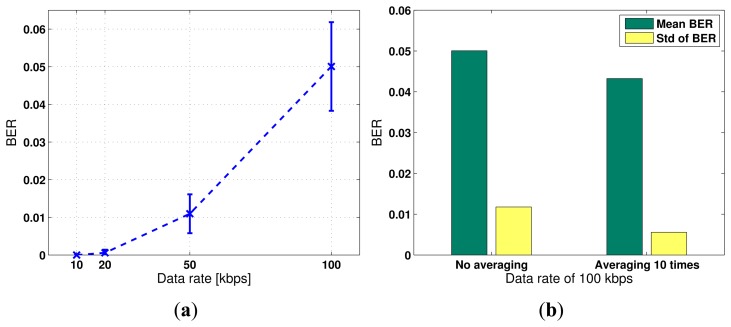
(**a**) Obtained bit error rate *versus* data rate at *R* = 10, 20, 50, 100 kbps; (**b**) impact of noise reduction by time averaging.

The obtained bit error rates under the four data rates are summarized in Table 2. Each bit error rate is averaged over 40 independent trials. For each trial, the information stream consists of 1, 000 randomly generated binary bits. Both the mean and the standard deviation of BER is shown in the table and is plotted in Figure 6(a).

We study the effect of noise level in the data acquisition system on the communication BER. We average the received responses 10 times to reduce the white noise and compare the results from data acquisition without averaging. Figure 6(b) shows that the mean and the standard deviation of BER over multiple trials reduces to 4.33 × 10^−2^ and 5.55 × 10^−3^, respectively, as compared to 5.01 × 10^−2^ and 1.17 × 10^−2^, when the white noise is not suppressed by averaging. However, the improvement is not significant. This implies that the improvement of the signal-to-noise ratio by averaging is insignificant compared to that by applying the time reversal focusing technique.

### 3.4. Results from Experiment II

Next, we conducted experimental tests on a stainless steel pipe specimen of 1.5 meters in length, 115 mm in outside diameter and 6 mm in wall thickness. Our goals were to validate that our technique could be generalized to (1) pipes of different materials, dimensions and loading conditions; (2) sensors of different sizes and inter-sensor distances; and (3) different noise levels. Two PZT wafers (PSI-5A4E) were mounted on the surface of the pipe using cyanoacrylate adhesive, located 0.69 m apart. Each wafer was 10 mm long and 10 mm wide. The stainless steel pipe vertically stood on the floor, with the bottom end sealed with a rubber cap, which allows the pipe to hold water. Randomly generated bit streams at data rates of *R* = 10, 20, 50, 100 kbps were sent through the pipe specimen using the TR-PPM method under two testing scenarios: the pipe was empty (without water) and the same pipe was filled with water. Furthermore, we also tested the communication results when the time averaging (10 times) of received responses was employed when generating y(−*t*) for modulation.

**Figure 7.**
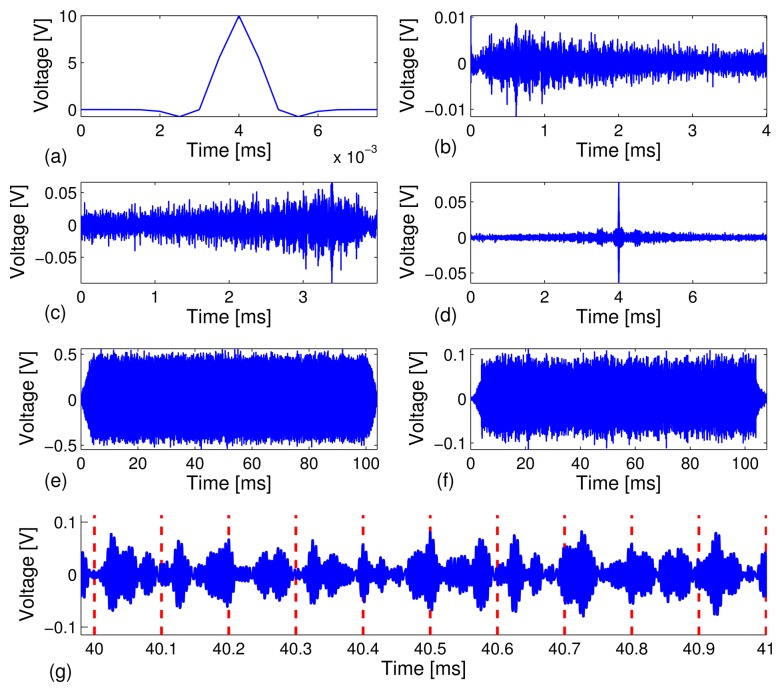
Snapshots of waveforms obtained experimentally from TR-PPM data communications on a stainless steel pipe (not filled with water) at a data rate of **10** kbps. (**a**) Gaussian pilot signal of 8 *μs;* (**b**) received response after channel sounding; (**c**) time reversal transmission; (**d**) received time reversal focused waveform; (**e**) modulated time reversal PPM waveform; (**f**) received information carrying waveforms; and (**g**) zoomed-in plot of (**f**). The red dashed lines indicate the starting positions of symbol frames. The transmitted bit stream in the time window of 40 ms and 41 ms is x[1 1 0 1 0 0 1 1 0 1 x].

**Figure 8.**
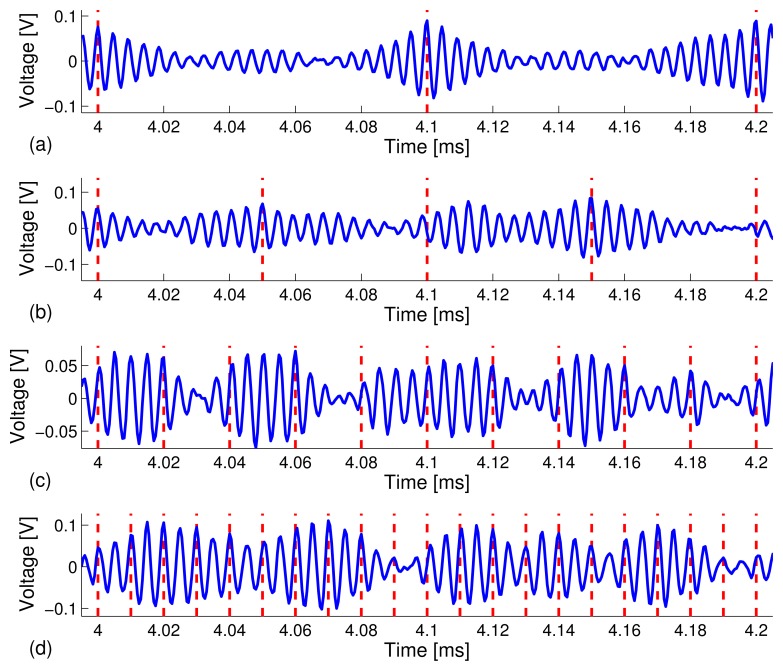
Snapshots of the received information carrying waveforms in the time window of 4.0 ms - 4.2 ms obtained from a stainless steel pipe (not filled with water). The data rates are (**a**) 10 kbps; (**b**) 20 kbps; (**c**) 50 kbps; and (**d**) 100 kbps.

Similar to the experimental results on the carbon steel pipe, we observed a complex channel sounding response on the stainless steel pipe (see Figure 7(b)) and a strong time reversal focused peak (see Figure 7(d)). However, we also noticed a higher level of sidebands around the time reversal focused peak compared to what we observed in the carbon steel pipe specimen in Experiment I (see Figure 3(d)). This phenomena are caused by the relatively small ratio of the transmitter/receiver distance (*D* = 0.69 meter) to the pipe diameter (*r* = 115 mm), *i.e.*, *D*/*r* = 690/115 = 6 for the stainless steel pipe setup, whereas the ratio is about *D*/*r* = 1500/70 = 21 for the configuration of the carbon steel pipe experiment in Experiment I. We note that elastic waves that propagate in pipes typically consist of longitudinal waves, torsional waves and flexural modes [45,46]. After excitation, different wave modes would appear as the waves travel along the pipe and interrogate the pipe. Since only one PZT receiving sensor was used at a single position on the pipe, many wave modes that were excited by the transmitting PZT sensor could not be captured, which, in turn, gave rise to a smaller focusing peak and larger sidebands after time reversal. We anticipate that using an array of PZT receivers could achieve a better time reversal focusing, because the use of a sensor array could capture more transmitted wave energy caused by various wave modes. Due to space limitation, the test results using sensor arrays will be reported elsewhere. Figure 7(g) depicts a zoomed-in plot of Figure 7(f) in the 40 ms - 41 ms time window, which includes the decoded 10-bit stream as x[1 1 0 1 0 0 1 1 0 1 x]. Figure 8 depicts snapshots of the received information carrying waveforms in the 4.0 ms - 4.2 ms time window for the four different data rates.

**Figure 9.**
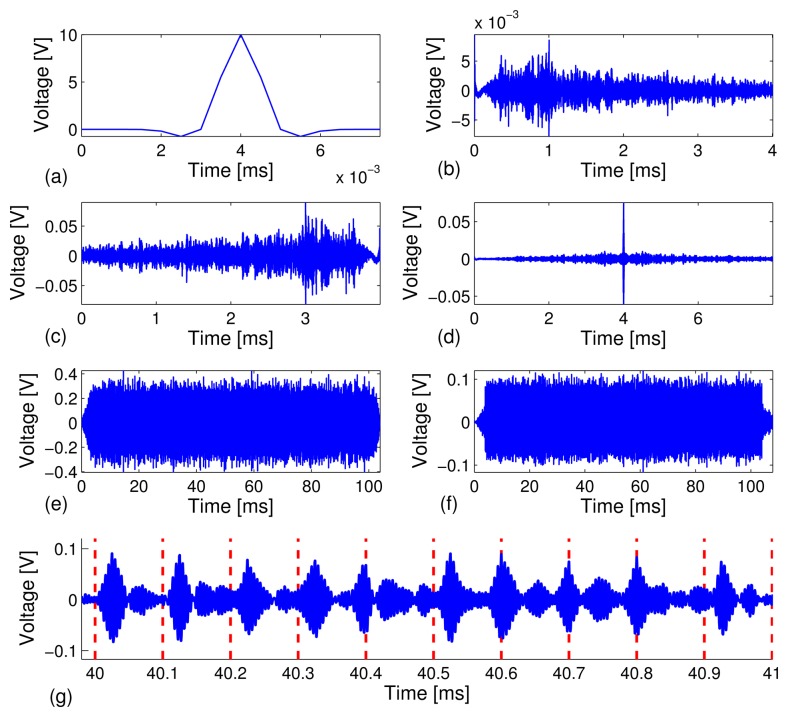
Snapshots of waveforms obtained experimentally from TR-PPM data communications on a stainless steel pipe filled with water at a data rate of 10 kbps. (**a**) Gaussian pilot signal of 8*μs*; (**b**) received response after channel sounding; (**c**) time reversal transmission; (**d**) received time reversal focused waveform; (**e**) modulated time reversal PPM waveform; (**f**) received information carrying waveforms; and (**g**) zoomed-in plot of (**f**). The red dashed lines indicate the starting positions of symbol frames. The transmitted bit stream in the time window of 40 ms and 41 ms is x[1 1 1 1 0 1 0 0 0 1 x].

We repeated the same experimental procedure on the stainless steel pipe specimen by filling the pipe with water. When filling the pipe with water, a few drops of detergent liquid were added to the water to release bubbles at the pipe-water interface inside of the pipe. The bubbles were observed accumulating at the water surface and released to air instead. Figure 9 depicts the snapshots of waveforms obtained during the TR-PPM data communication schemes similar to what was presented in Figure 7 for the test scenario where no water is in the pipe specimen. Figure 9(d) and (g) show that the waveforms have less sidebands compared with Figure 7(d) and (g), which implies less interference caused by the sidebands of the time reversal focused response. The bit stream turns out to be more distinguishable when the pipe is filled with water. Our hypothesis is that water suppresses certain guided wave modes in the pipe (such as flexural modes), due to the water damping effects [47]; thus, the adverse effect on time reversal focusing, due to a short inter-transducer distance, becomes less significant.

**Figure 10.**
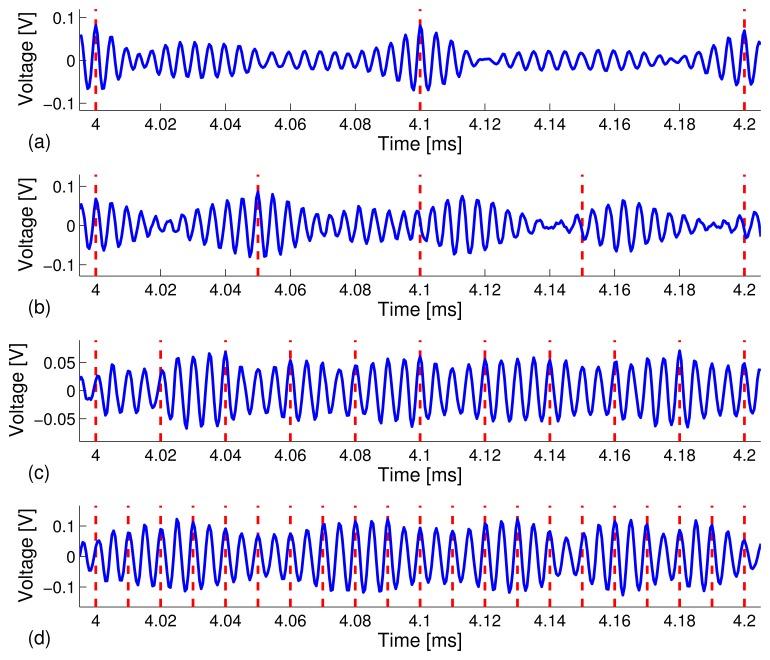
Snapshots of the received information carrying waveforms in the time window of 4.0 ms - 4.2 ms obtained from a stainless steel pipe specimen filled with water. The data rates are: (**a**) 10 kbps; (**b**) 20 kbps; (**c**) 50 kbps; and (**d**) 100 kbps.

**Figure 11.**
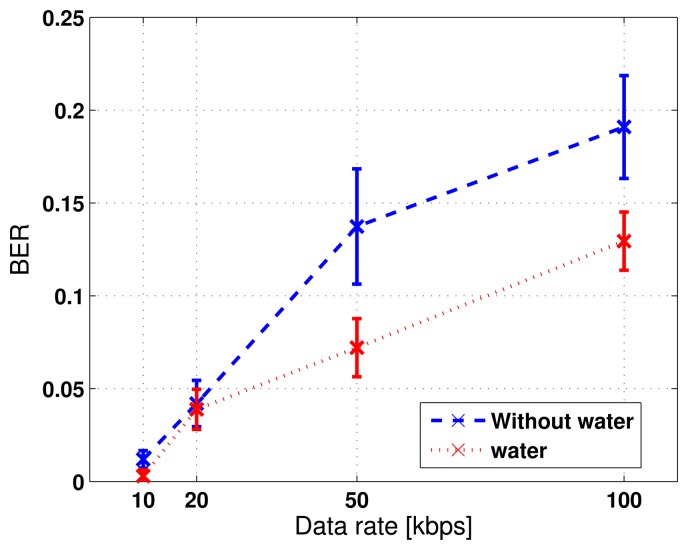
Obtained bit error rate *versus* data rates of *R* = 10, 20, 50, 100 kbps for stainless steel pipe specimen.

As a result, we achieved a lower BER for TR-PPM communications on the pipe that was filled with water at the data rates of *R* = 10, 20, 50, 100 kbps, compared to the scenario when the pipe was not filled with water. Next, Figure 10 depicts the snapshots of the received information carrying waveforms in the 4.0 ms - 4.2 ms time window under data rates of *R* = 10, 20, 50, 100 kbps, respectively. The BER results are summarized in Table 3 and plotted in Figure 11.

Furthermore, we also utilized time averaging (by a factor of 10) to reduce noise level in the received responses for both scenarios (water filled and no water) on the stainless steel pipe specimen. The improvement due to time averaging is insignificant, as shown in the Table 4, where the BER results for the case of non-averaging and that of averaging at a 100 kbps data rate are summarized. The results are what we have expected, because Equation (29) reveals that the bit error rate depends on the noise power and the inter-symbol interference. Averaging will increase the signal-to-noise ratio by reducing the noise power, but has a limited effect on the side-band level that characterizes the inter-symbol interference. However, it is expected that time averaging would be effective in the environment when the noise level is relatively high.

## 4. Conclusions and Discussions

In this paper, we present the theoretical background and experimental studies of the time reversal-based timing acquisition and pulse position modulation method for pulsed wideband elastic wave communication on steel pipes. In this method, the focusing properties of time reversal are used to exploit the strong dispersion and multi-modality of elastic channels to enable accurate timing acquisition and symbol demodulation. As a result, a single finger receiver combined with a single sliding correlation detector suffices for the system to rapidly obtain the starting time. Once the timing offset between the transmitting sensor and the receiving sensor is obtained, modulated symbols are transmitted, received and demodulated to recover the original information symbol. Therefore, complicated channel estimation algorithms are not necessary, due to time reversal, and the computational efficiency is improved. From a hardware perspective, sophisticated circuit implementation is not required.

We conducted experimental studies on steel pipe specimens using piezoelectric PZT sensors in a laboratory environment. The experimental results demonstrate that the proposed TR-PPM scheme achieves robust communications at data rates of 10 kbps and 20 kbps on steel pipe specimens in spite of the severe channel dispersion and multi-modality of the elastic channels. To achieve higher data rates, more complicated processing techniques, such as adaptive equalization or error control coding should be used in conjunction with the time reversal approach. In addition, we note that the system has been tested over a rather small distance (a maximum of 1.5 meter), due to limitation of the specimen's dimension. The estimated attenuation factor is about 26 dB/m, as described in Section 3.3. In general, as they are propagating, guided waves may experience attenuation or reduction in amplitude. This scenario occurs mainly in two situations or a combination of both [48]: *i.e.*, material absorption of wave energy due to viscoelastic materials in the medium [49] and energy leakage into a different medium at the boundaries [50]. The efficacy of the proposed TR-PPM over distances that are an order of magnitude greater than the ones tested in our experiments is influenced by the attenuation factor, which, in turn, depends on the excitation frequency, the material property, the type of generated wave modes, *etc.* Although not tested in a more realistic environment on sub-sea structures or pipelines, the proposed scheme is expected to perform well over a longer distance in a larger scale, for example, a 50 meter range if a 60 dB signal-to-noise ratio (SNR) loss is allowed, due to wave attenuation, based on the observed 26 dB/m attenuation factor.

## Figures and Tables

**Table 1.** Test parameters for TR-PPM communications.

**Data Rate (kbps)**	**Frame Time (μs)**	**# of bits Transmitted**	**Sampling Frequency (MHz)**	**Center Frequency (kHz)**	**Bandwidth (kHz)**	**Time Shift (μs)**	**# of Trials**
10	100	1, 000	2	250	500	25	40
20	50	1, 000	2	250	500	12.5	40
50	20	1, 000	2	250	500	5	40
100	10	1, 000	2	250	500	2.5	40

**Table 2.** Bit error rate (BER) obtained from experiments.

	**Data Rate (kbps)**	**10**	**20**	**50**	**100**
BER	mean	0	5.75 × 10^−4^	1.09 × 10^−2^	5.01 × 10^−2^
	std	0	8.33 × 10^−4^	5.15 × 10^−3^	1.17 × 10^−2^

**Table 3.** Obtained bit error rate (BER) for stainless steel pipe specimen.

	**Data rate (kbps)**	**10**	**20**	**50**	**100**
Without Water	mean	1.195 × 10^−2^	4.202 × 10^−2^	0.137	0.190
	std	4.716 × 10^−3^	1.243 × 10^−2^	3.103 × 10^−2^	2.771 × 10^−2^

With Water	mean	2.925 × 10^−3^	3.887 × 10^−2^	7.212 × 10^−2^	0.129
	std	3.408 × 10^−3^	1.078 × 10^−2^	1.565 × 10^−2^	1.565 × 10^−2^

**Table 4.** Impact of Noise Reduction by Time Averaging at 100 kbps.

	**BER**	**No Averaging**	**Averaging 10 Times**
Without Water	mean	0.190	0.181
	std	0.027	0.015

With Water	mean	0.129	0.095
	std	0.015	0.012
